# My Transformation as a Trainee in a Halstedian Surgical Residency: A Tribute to My Mentors

**DOI:** 10.1093/asjof/ojz027

**Published:** 2019-10-07

**Authors:** Andrew N Kornstein

**Affiliations:** Dr Kornstein is a plastic surgeon in private practice in Greenwich, CT, and in Wilton, CT

How often in our lives are we fortunate enough to identify a person or experience that becomes a prevailing current for the rest of our lives—a soulmate, a financial windfall or disaster, spiritual enlightenment, stardom, working as part of a unit (eg, in the military), or finding a calling, such as medicine? For me, it was my experience as one of eleven Ivy League interns in the traditional Halstedian every other night pyramid surgical residency at Roosevelt Hospital (RH) in New York City, which shaped my evolution as a surgeon.

What made this experience so powerful? I believe it was a combination of the tradition and history of RH as well as the brilliant individuals who shaped and guided interns and residents at this location. Such an experience stays with you and becomes a template for other challenges in your life. I know there are others in this profession who had similar training experiences and share these feelings.

## THE TRADITION

RH opened its doors in 1871, a few years after the Civil War ended.^1^ RH, a surgical hospital, was the workplace of William Halsted, MD, the father of modern surgery and of surgical residency. Dr Halsted organized the outpatient “dispensary” at RH in 1881 and served as its director until 1886.^1^ There, Halstead pioneered the use of peripheral nerve cocaine anesthesia for minor surgical operations. In 1888, Charles McBurney, MD, assumed responsibility for the design and planning of the Syms Operating Theatre, which opened in 1892.^1^ The Syms Operating Theatre still is standing on the southwest corner of 59th and 9th Avenue was widely hailed as a model of its kind and one of the finest in the world. While at RH in 1893, Dr McBurney also described McBurney’s Point, for diagnosis of acute appendicitis.

Robert F. Weir, MD, developed the Weir Excision in rhinoplasty and was among the first to adopt, urge, and teach Lister’s methods of antisepsis. At RH, Dr Weir also performed and documented the first total removal of an unequivocal brain tumor in the United States.^1^ Thomas L. Bennett, MD, introduced the nitrous oxide–ether sequence and went on to design the Bennett Inhaler, which was considered standard equipment in anesthesia administration for many years after.

**Figure 1.**
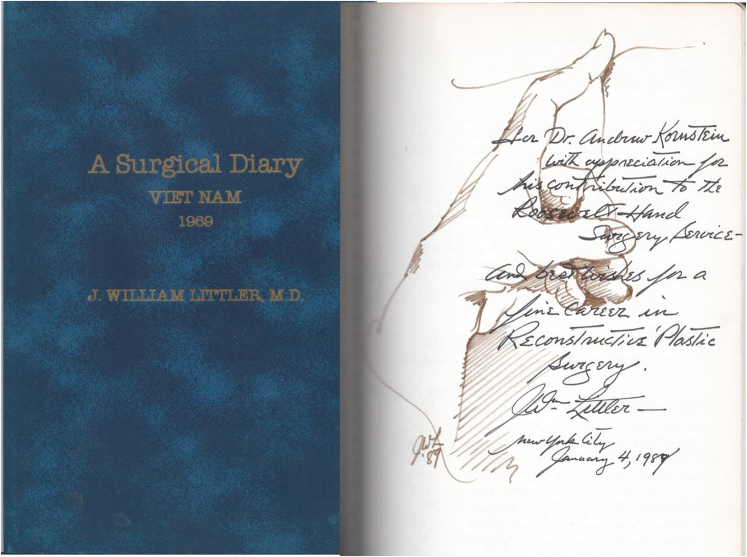
Littler’s Vietnam Diary, inscribed with one of his legendary hand drawings and “best wishes for a fine career.”

RH was the first location at which medical students received instruction (1914); Columbia University Physicians & Surgeons was across the street on 9th Avenue, between 58th and 59th Street. Finally, J. William Littler, MD—the father of hand surgery—established the Hand Center at RH, where he practiced this surgical subspecialty. I thank Saul Kaplan, MD, hand surgery fellow, for asking Dr Littler to inscribe a copy of his Vietnam Diary for me (Figure 1). I will always cherish this piece of surgical history.

## THE PEOPLE

I wish to deeply thank the teachers, leaders, and mentors—now deceased—who apprenticed my colleagues and me during our time at RH. These surgeons motivated us to work hard enough to “compete for a spot,” as the number of house staff was reduced each year, while still fostering and supporting a cooperative, functional team. They did this all while instilling the requisite skills and responsibilities of a physician-surgeon. I regret to say that I was not aware enough, or perhaps awake enough, at that time to understand the gift these men and women gave me.

As interns and residents, we have our jobs to do and our lessons to learn. What distinguishes us from other professionals is that we have personal responsibility for the well-being of those under our care. From examinations to procedures, our skills and personality come together to influence the outcomes of our patients. We are taught to work in a system where all contribute: technicians, nurses, and doctors from other specialties. Medicine is impossible to practice alone—and this is especially true for operating-room surgery.

With extensive, prolonged exposure to clinical disease presentation, treatment, and outcomes, we develop a medical “gut instinct.” In this regard, time spent as interns and residents in the hospital constitutes a golden period of our medical apprenticeship. This is all part of the slow, subliminal molding that must occur for a doctor to be able to make life-altering decisions each day. For the profession and for those under our care, we develop feelings of respect, devotion, and responsibility, and we evolve as physicians alongside the changing demands of the healthcare industry.

There is so much more to assimilate than merely the mechanics of medicine and surgery. As trainees in our twenties, we are pulled in so many directions personally and professionally; it is no wonder most of us fail to appreciate what is happening to us during this time. Nevertheless, our teachers and mentors leave indelible impressions on us as we grow more deeply in love with medicine until the day we choose to put our scalpels down—or have them pulled from our surgically gloved hands.

It is hard, perhaps impossible, to envision the person one would be today without the experiences of yesterday. Herein, I wish to honor those who banded together to raise me from a Cornell University medical student to a doctor: devoted, conscientious, and prepared for the challenges that lie ahead. Thank you all, I now fully appreciate what you did then to make me who I am today.

- Dr Wichern (“The Wich”), surgeon-in-chief, a brilliant and confident surgeon who stood by scrubbed with arms folded in the corner of the operating room overseeing our surgeries and at-the-ready to bail us out when we got in trouble. No one knew the human body better; he helped us become calmer and better prepared to make surgical decisions.
- Dr Marks, the most exacting, dedicated, and accurate physician-surgeon I have known. Pre-rounding and having Dr Marks write “as well described above” was truly a reward that kept me going through the hard times. With no surgeon did I spend more time in the operating room. Dr Marks was the embodiment of someone devoted to a career in medicine.
- Dr Moore, a giving and enthusiastic teacher. He was one of us in so many ways. He never lost the enthusiasm of his days as a trainee, and he enjoyed nothing more than teaching residents.
- Dr Dailey, also from Cornell Med, former president of the Colorectal Society. He took me through my first surgery as an intern, an inguinal hernia. He was imposing in size and in personality. He required of us the skills, focus, and demeanor of medical professionals.
- Dr Yeoh, who was unparalleled with regard to his diverse surgical practice and his confidence in the house staff. He allowed us to be ourselves, gradually giving us more responsibilities and fostering our decision making and procedural growth. Letting him down was letting ourselves down.
- Dr Beal, breast surgeon extraordinaire, who explained to me at the sink—scrubbing for a surgery—that being a surgeon was an identity, not a job or a profession. At the time, I could not comprehend fully what he was saying. I now understand that, like him, I am a surgeon first; it is what I work at the hardest and enjoy the most.

## CONCLUSIONS

The traditions of RH and the men and women who operated there greatly influenced my life and enabled me to feel the thrill of being a confident and well-schooled surgeon and to positively touch the lives of so many. Is there anything more important in our brief time on earth than helping other human beings? My mentors at RH empowered me with the skills and direction to be able to seek and find that answer. I can only hope that future generations of physicians will be exposed to the same traditions and lessons that helped give my life its professional purpose.

## Disclosures

The author declared no potential conflicts of interest with respect to the research, authorship, and publication of this article.

## Funding

The author received no financial support for the research, authorship, and publication of this article.
